# REHABILITATION INTERVENTIONS FOR NEUROPATHIC PAIN: A SYSTEMATIC REVIEW AND META-ANALYSIS OF RANDOMIZED CONTROLLED TRIALS

**DOI:** 10.2340/jrm.v56.40188

**Published:** 2024-08-05

**Authors:** Emmanuel BÄCKRYD, Nazdar GHAFOURI, Björn GERDLE, Elena DRAGIOTI

**Affiliations:** Pain and Rehabilitation Center, and Department of Health, Medicine and Caring Sciences, Linköping University, Linköping, Sweden

**Keywords:** chronic pain, interdisciplinary, multimodal, neuropathic pain, outcome, rehabilitation

## Abstract

**Objective:**

Rehabilitation interventions for chronic pain typically include education, cognitive behavioural therapy, and exercise therapy, or a combination of these. A systematic review and meta-analysis of rehabilitation interventions for neuropathic pain was conducted.

**Design:**

Randomized controlled trials were identified in PubMed, EMBASE, Cochrane Central Register of Controlled Trials, and PsycINFO databases from inception up to 3 March 2022.

**Subjects/Patients:**

Adults with chronic (> 3 months) neuropathic pain.

**Methods:**

Primary outcomes were pain intensity, pain-related disability, and work participation. Secondary outcomes were quality of life, emotional strain, insomnia, and adverse outcomes, according to VAPAIN guidelines. Analyses were made post-intervention, which was defined as the assessment point immediately following the intervention or at the first-time measurement conducted after the intervention period.

**Results:**

In total, 15 studies (total population, *n* = 764) were incorporated. Most common interventions were cognitive behavioural programmes including acceptance and commitment therapy (*n* = 4), mindfulness-based interventions (*n* = 5), and yoga (*n* = 2). Psychological interventions reduced both pain intensity (SMD –0.49, 95% CI –0.88 to –0.10) and pain-related disability (SMD –0.51, 95% CI –0.98 to –0.03), whereas other interventions had an effect on pain intensity but not on pain-related disability.

**Conclusion:**

Rehabilitation interventions, and psychological interventions in particular, seem to be of value for patients with chronic neuropathic pain.

## LAY ABSTRACT

When chronic pain cannot be alleviated by medicines or by other “passive” interventions, it is still possible to help the patient live a good life despite the pain by using rehabilitation interventions such as, e.g., education, cognitive behavioural therapy, exercise therapy, or a combination of these. We have evaluated the literature concerning the effect of rehabilitation interventions on chronic neuropathic pain in adults. We found 15 studies encompassing a total of 764 patients. The most common interventions were cognitive behavioural programmes including acceptance and commitment therapy (4 studies), mindfulness-based interventions (5 studies), and yoga (2 studies). Such psychological interventions reduced both pain intensity and pain-related disability. Other interventions had an effect on pain intensity but not on pain-related disability. However, due to lack of data, we could assess only short-term effects (< 6 months). More studies are needed to further assess the effects of rehabilitation interventions for chronic pain patients.

Neuropathic pain is defined as pain caused by a lesion or disease affecting the somatosensory nervous system (1). About 7% of the population suffers from pain with neuropathic characteristics (2, 3). In the coming decades, it is possible that the prevalence of neuropathic pain will increase due to several factors, such as an ageing population (4), an increased number of cancer survivors suffering from treatment-induced neuropathy (5), and the metabolic syndrome epidemic (6–8) as 1 in 5 diabetic patients develops neuropathic pain (9). There are evidence-based guidelines for the pharmacological treatment of neuropathic pain, but even when the guidelines are followed many patients do not experience adequate pain relief, defined as 30% or 50% pain reduction (10).

The pharmacological treatment of neuropathic pain is based mainly on 2 groups of medicines: some antidepressants (mainly amitriptyline and duloxetine) on the one hand, and α_2_δ ligands (gabapentin and pregabalin – collectively known as gabapentinoids) on the other hand. Even when these first-line medicines are prescribed adequately, only a minority of patients get substantial pain relief, numbers needed to treat (NNT) for > 30–50 % pain relief being in the range of 4–8 for each of these 4 drugs (10). In clinical practice, and because of its safety profile, transcutaneous nerve stimulation is often used (11). In a minority of severe cases, advanced invasive methods such as spinal cord stimulation (SCS) (12) or intrathecal analgesia (13) can be used, but for a majority of neuropathic pain patients adequate pain relief will not be achieved.

Chronic pain patients can be offered participation in interdisciplinary pain rehabilitation programmes (IPRP), in which a team consisting of professionals from different disciplines (e.g., psychologists, physiotherapists, physicians, and occupational therapists) work in a synchronized manner to help the patient from a rehabilitative and holistic perspective. Simply put, if the pain cannot be alleviated by using analgesic medicines, there are still ways in which the patient can be helped to live an active life despite pain. Rehabilitation interventions in the context of chronic pain typically comprise education on pain and coping skills, cognitive behavioural therapy-based interventions (CBT), and exercise therapy. Compared with many other chronic pain conditions, little research has been conducted on how such interventions may benefit patients suffering from chronic *neuropathic* pain conditions (14–16). Among chronic pain conditions, neuropathic pain patients tend to participate in rehabilitation interventions to a lesser extent than other pain patients, such interventions having been developed mainly for other pain conditions such as fibromyalgia or chronic back pain (14, 17). Some clinicians also seem to think that neuropathic pain patients are “too complicated” for rehabilitation. Hence, the place of IPRP, or its components, in the treatment of neuropathic pain needs to be substantiated by more research. As there seems to be an ongoing shift towards more of a rehabilitation perspective in neuropathic pain treatment recommendations (15, 17), and because there is a lack of systematic reviews regarding this issue, we have conducted a systematic review and meta-analysis on rehabilitation interventions for neuropathic pain.

## METHODS

### Protocol registration

The methods and planned analyses of this systematic review were pre-registered on 21 March 2022 at PROSPERO (CRD42022311644). We also followed the PRISMA 2020 guidelines (Table SI) (18). All deviations from the pre-registered procedures and analysis plans are clearly marked in the manuscript.

### Literature search

Two investigators (ED, EB) independently performed a systematic search for relevant studies. Studies were initially identified by title and abstract in the PubMed, EMBASE, Cochrane Central Register of Controlled Trials (CENTRAL), and PsycINFO databases, using the terms “neuropathic pain”, “neuropathy”, “neuralgia”, “rehabilitation”, “psychotherapy”,” physiotherapy”, “vocational rehabilitation”, “physical training”, and “randomized controlled trial” as well as any possible combinations of these terms, from inception to 3 March 2022. Details of the search strategy are given in Box S1. The search was complemented by a manual review of reference lists of relevant publications to find additional studies on the topic. Attempts have been made to contact the original authors for unsupported data or ongoing studies. Discrepancies between the 2 investigators during the selection process were resolved by a third investigator (BG).

### Eligibility criteria

We followed the Population/Patients/Problem-Intervention-Comparison-Outcome-Study (PICOS) process to select the primary studies included in this systematic review.

### Population/patients/problem

Patients were adults (aged 18 years and older) with chronic neuropathic pain. The pain had to be chronic, i.e., have lasted at least for 3 months as diagnosed by a clinician or using any recognized diagnostic criteria. Additional inclusion criteria for chronic neuropathic pain conditions were: (*i*) peripheral neuropathic pain: trigeminal neuralgia, after peripheral nerve injury (trauma or surgery), polyneuropathy, postherpetic neuralgia, radiculopathy, painful diabetic neuropathy, cancer-related neuropathy, or peripheral polyneuropathy of other aetiologies, for example toxic (alcohol, drugs, etc.) and (*ii*) central neuropathic pain: associated with spinal cord injury, brain injury, post-stroke pain, multiple sclerosis, or phantom limb pain.

Exclusion criteria in primary studies were if they were considered to be in pain for less than 3 months (i.e., acute, and subacute pain), experimental pain, complex regional pain syndrome (CRPS), no control group, chronic musculoskeletal pain, pharmacological and surgical or invasive therapies. Whenever both subacute and chronic pain populations were included in the same study, we retained the study only if at least 75% of the patients were diagnosed with chronic pain.

### Interventions

This study investigated comparisons of any type of rehabilitation interventions. The following interventions were studied: psychological interventions; educational and self-management interventions; work-related interventions; physical interventions; physical activity/exercise interventions; interdisciplinary and multidisciplinary pain interventions consistent with the bio-psycho-social model of pain (19). Comparators were interventions such as nerve blocks, or inactive control such as placebo, treatment as usual (TAU), no intervention, or another active intervention. The presence or absence of concomitant medication was not part of the investigation.

### Outcomes

The primary outcomes included *pain intensity* (e.g., by visual analogue scale (VAS), or numerical rating scale [NRS]); *function or pain-related disability* (e.g., brief pain inventory [BPI], pain disability index [PDI]); *work participation* (e.g., as the rate of time until first return to work after sickness by self-report or based on information collected from organizational or system record measurements). Additional secondary outcomes were quality of life (QoL), emotional strain, insomnia, adverse outcomes, etc., according to VAPAIN guidelines (20).

### Study selection

We included only randomized controlled trials (RCTs) with a parallel study design written in English or Swedish, and no publication year, setting, or time frame restrictions were applied. For crossover studies, we recorded the results and randomization before the first washout period.

### Risk of bias

The revised Cochrane Risk of Bias (RoB 2.0; https://methods.cochrane.org/bias/resources/rob-2-revised-cochrane-risk-bias-tool-randomized-trials) tool was used by two of us (EB and NG) to evaluate the methodological quality of the studies included (21). The RoB 2.0 tool includes 7 criteria: random sequence generation, allocation concealment, blinding of patients and personnel, blinding of outcome assessment, incomplete data outcomes, selective outcome reporting, and other biases. The overall risk of bias for each study was graded as low, high, or some concerns. Any dispute was settled through discussion. If the discussion still failed, a third author decided (ED).

### Data management and extraction

All citations obtained using the search strategy were imported into Endnote X9 (https://endnote.com/), and duplicates were deleted using the “Find Duplicates” function. A second approach for duplicates were done manually using Microsoft Excel (Microsoft Corp, Redmond, WA, USA). Two of the authors (EB and NG) independently extracted data. Any disagreement was resolved by discussion until consensus was reached or by consulting a third investigator of our team (ED). The following data were extracted: author, year of publication, study period, type of neuropathic pain, diagnostic criteria, total number of patients included in the study, number of patients per arm, number or rates of dropouts per arm, mean age of patients, percentage female, treatment format: individual or group, hours of treatment, number of treatment sessions, type of outcome measure, and data that were used to calculate the effect size (mean, SD or median, IQR, effect sizes, ORs, RRs). The primary endpoint for all analyses was post-intervention, which we defined as the assessment point immediately following the intervention or at the first-time measurement conducted after the intervention period.

### Assessing certainty of evidence

The certainty of evidence for the primary outcome was assessed using the Grading of Recommendations Assessment, Development, and Evaluation (GRADE) approach (22).

### Data analysis

The meta-analysis was conducted using Stata 17.0 (StataCorp LLC, College Station, TX, USA). Pooled effect sizes and corresponding 95% confidence intervals (CI) for each outcome were calculated using the “metan” command. To account for potential heterogeneity among the included studies, random-effects models were selected (23). For continuous outcomes, the effect size metric was chosen as either the standardized mean difference (SMD) or mean difference (MD). Heterogeneity was assessed with the “metan” command, and the I² statistic quantified the degree of heterogeneity across studies. I² values of 25%, 50%, and 75% indicated low, moderate, and high levels of heterogeneity, respectively (24, 25). Subgroup analyses were conducted to explore potential sources of heterogeneity, stratifying by predefined variables such as intervention type, whenever applicable. Funnel plots and Egger’s regression test were used to assess publication bias (26) when the included studies were ≥10 (27). Additionally, meta-regression analyses were performed to explore the impact of variables on effect sizes (28). All statistical tests were two-tailed, and statistical significance was set at *p* < 0.05.

### Difference between protocol and review

Due to a lack of data, we focused solely on short-term outcomes. Consequently, unlike the protocol published in PROSPERO (CRD42022311644), the present paper did not include data regarding long-term effects (beyond 6 months). Additionally, planned sensitivity analyses, such as restricting the analysis to high-quality studies, conducting leave-one-out meta-analyses, and performing subgroup analyses within subgroups of chronic neuropathic pain (differentiating between central and peripheral neuropathic pain), gender, and age (comparing women vs men and younger ages [≤ 49 years old] vs older ages [≥ 50 years old]), as well as assessing combined interventions vs single interventions, were not conducted due to insufficient reporting of data.

## RESULTS

### Search results

**Fig. 1:**
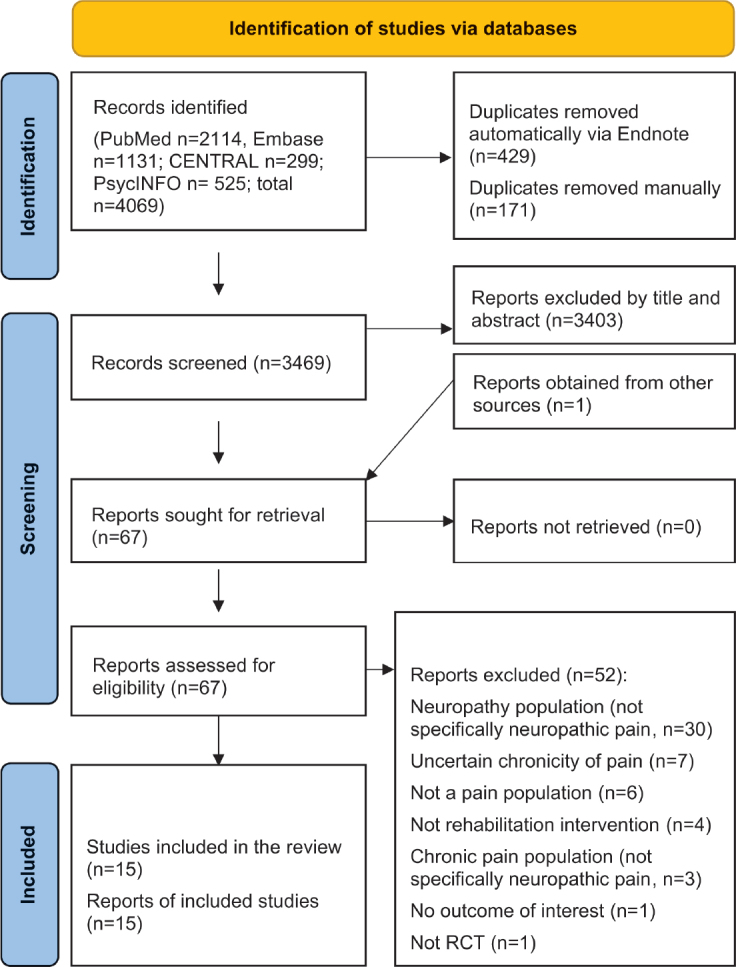
Study selection flowchart.

In our electronic search, a total of 4,069 studies were initially identified. Among these, 2,114 were discovered via PubMed, 1,131 through EMBASE, 299 from CENTRAL, and 525 via PsycINFO (as illustrated in Fig. 1). We carefully compared these studies for duplicates, considering matching author names, publication years, titles, and abstracts. After eliminating 600 duplicates, we were left with 3,469 unique records, which underwent initial screening. One publication was also obtained from reference lists of relevant studies.

Subsequently, we thoroughly reviewed 67 full-text studies that appeared to be potentially eligible, ultimately incorporating 15 of them into our analysis (29–43). Most publications (*n* = 37) were excluded primarily due to specific reasons such as wrong population (not specifically neuropathic pain) and uncertain chronicity of pain. A comprehensive list of all the excluded studies can be found in Table SII.

### Characteristics of included studies

Table I provides an overview of the characteristics of all 15 included studies. All but 2 studies (30, 34) followed a 2-arm, randomized, controlled trial design. Four studies were conducted in the United States, followed by 3 in Turkey, 2 in Canada and United Kingdom, and 1 in Australia, China, India, and the Netherlands.

**Table I:** Characteristics of included studies

Author, year	Study design	Country	Type of neuropathic pain	Sample size	Sample analysed	Intervention group	Control group(s)	Mean age (SD)	Female (%)	Intervention length, frequency, duration	Intervention home practices	Outcomes
Allison, 2002	Randomized controlled trial (3 arms)	Australia	Cervicobrachial pain syndrome	30	30	Neural tissue manual therapy (NTMT; *n* = 10)	Control group (CG; *n* = 10): Waiting list	MTMT: 50.0 (19.5) AT: 61.0 (8.5)	66.7	8 weeks, NR	A series of home mobilization	Pain intensity (VAS 0–10; MPQ total), Functioning (NPQ)
						Articular treatment (non-specific manual therapy; AT; n = 10)		CG: 52.5 (10.0)				
Gok Metin, 2017	Open-label randomized controlled trial (2 arms)	Turkey	Painful diabetic neuropathy	46	46	Aromatherapy massage (AM; *n* = 21)	Control group (CG; *n* = 25): Usual care	AM: 54.3 (8.8) CG: 57.2 (9.7)	76.1	4 weeks, 3 massage sessions of 30 min massage, 6 h in total	NR	Pain intensity (VAS 0–10), Quality of life (NePIQoL)
Heutink, 2012	Randomized controlled trial (2 arms)	Netherlands	Spinal cord injury neuropathic pain	61	60	Cognitive behavioural treatment programme (CBTP; *n* = 31	Control group (CG; *n* = 30): Waiting list	All: 58.8 (11.4)	36.1	10 weeks, 10 sessions of 3 h, 30 h in total	NR	Pain intensity (CPGQ 0–100), Functioning (CPGQ 0–100) Mood (HAD), Participation in activities (UAL), Life satisfaction (LSQ)
Hearn, 2018	Randomized controlled trial (2 arms)	UK	Spinal cord injury neuropathic pain	67	43	Mindfulness based pain management (MBPM online; *n* = 36)	Control group (CG; *n* = 31 ): Psychoeducation online	MBPM: 43.8 (8.7) CG: 45.2 (12.2)	54	8 weeks, 6 per week, 20 min	NR	Pain intensity (NRS 0–10) Depression (HADS-14-D 0–21) Anxiety (HADS-14-A 0–21) Quality of Life (WHOQoL-BREF 0–100) Catastrophizing (PCS-13 0–52)
Higgins, 2022	Randomized controlled trial (2 arms)	USA	Painful diabetic neuropathy	47	42	Cognitive behavioural therapy (CBT; *n* = 23)	Control group (CG; *n* = 24): Diabetes education	CBT: 63.8 (9.1) CG: 60.9 (7.6)	6.4	10 weeks, 10 sessions of 1 h per week, 10 h in total	NR	Pain intensity (VAS 0–10, NPS), Functioning (MPI-I), Depression (BDI), Quality of life (SF-36 Physical Health Scale and Mental Health Scale 0–100), Sleep quality (PSQI)
Izgu, 2020	Randomized controlled trial (3 arms)	Turkey	Painful diabetic neuropathy	77	69	Progressive muscle relaxation (PMR; *n* = 28)	Control group (CG; *n* = 24): attention control education	PMR: 64.2 (8.1) MM: 61.6 (8.0)	53.6	12 weeks, 50 min training session, 28.8 h in total	20 min daily MM or PMR at home	Pain intensity (VAS0-10), Fatigue (FACIT-F), Quality of life (NePIQoL)
						Mindfulness meditation (MM; *n* = 25)		CG: 64.1 (6.6)				
Kaur, 2020	Randomized controlled trial (2 arms)	India	Spinal cord injury neuropathic pain	44	42	Mental imagery (MI; *n* = 22)	Control group (CG; *n* = 22): 15 min arithmetic tasks+15 min listening to music	MI: 31.6 (10.7) CG: 29.3 (10.1)	23.8	4 weeks, 30 min per day, 5 days per week, 10 h in total	NR	Pain intensity (NRS 0–10), Functioning (BPI-activity, mobility), Mood (BPI-mood), Productivity (BPI-work), Satisfaction (BPI-relation), Sleep (BPI-sleep)
Knoerl, 2018	Multicenter, randomized controlled trial (2 arms)	USA	Painful chemotherapy-induced peripheral neuropathy (CIPN)	60	50	Cognitive behavioural therapy (CBT online; *n* = 30)	Control group (CG; *n* = 30): Usual care	CBT: 58.9 (9.3) CG: 63.4 (8.4)	75.0	8 weeks, 10 online modules	NR	Pain intensity (NRS 0–10), Functioning (PROMIS -interference), Impression of change (PGIC score ≥ 5), Quality of life (QLQCIPN20)
Knoerl, 2021	Randomized controlled trial (2 arms)	USA	Painful chemotherapy-induced peripheral neuropathy (CIPN)	45	37	Yoga programme (Y; *n* = 29)	Control group (CG; *n* = 16): Waiting list	Y: 55.3 (31.9) CG: 58.7 (31.7)	95.5	8 weeks, individual yoga sessions of 45 min	Livestreamed Zoom class (due to pandemic, after the first 21 patients)	Pain intensity (worst NRS 0–10), Functioning (PROMIS -interference; PROMIS-Physical function), Sleep (PROMIS-sleep-related impairment), Anxiety (PROMIS-anxiety), Fatigue (PROMIS-fatigue), Depression (PROMIS-depression)
Meize-Grochowski, 2015	Randomized controlled trial (2 arms)	USA	Postherpetic neuralgia	31	27	Mindfulness meditation (MM; *n* = 16)	Control group (CG; *n* = 15): Usual care	MM: 55.3 (31.9) CG: 58.7 (31.7)	55.6	6-weeks, 1–2 times per day, 3–15 min+an individual, 1-h session, 83 min in total	No	Pain intensity (SF-MPQ-2 0–10; RAND36-Pain), Functioning (RAND36), Anxiety (STAI), Depression (CES-D), Quality of life (RAND36)
Nathan, 2017	Randomized controlled trial (two arms)	Canada	Painful diabetic neuropathy	66	63	Mindfulness-based stress reduction (MBSR; *n* = 33)	Control group (CG; *n* = 33): Waiting list	MBSR: 59.7 (9.1) CG: 59.8 (8.7)	56.0	8 weeks, 2.5 h weekly sessions and midway through the course a 6 h weekend session, 26 h in total	NR	Pain intensity (BPI 0–10), Functioning (BPI -interference), Mood (POMS2A), Stress (PSS), Catastrophizing (PCS), Depression (PHQ-9), Quality of life (SF12)
Scott, 2021	Randomized controlled trial (2 arms)	UK	Painful HIV peripheral neuropathy	38	27	Acceptance and commitment therapy (ACT online; *n* = 25)	Control group (CG; *n* = 13): Waiting list	ACT: 55.8 (5.7) CG: 56.0 (6.2)	23.7	8 weeks, 45–60 min per session, 12 online sessions, 9–12 h in total	NR	Pain intensity (BPI 0–10), Functioning (BPI -interference, Work and social adjustment WSAS), Pain acceptance (CPAQ-8), Depression (PHQ-9)
Toth, 2014	Randomized controlled trial (2 arms)	Canada	Miscellaneous types of neuropathic pain	54	39	Exercise (EX; *n* = 28)	Control group (CG; *n* = 26): Education programme	EX: 54.8 (6.7) CG: 55.4 (7.0)	59.3	24 weeks, 2 h instruction from kinesiologist each month	Exercise at home 3–5 times weekly	Pain intensity (VAS 0–100), Functioning (KPS), Depression (HAD-D), Anxiety (HAD-A), Sleep (MOSSS), Quality of life (EQ5D)
Yildirim, 2022	Randomized controlled trial (2 arms)	Turkey	Lumbar radicular pain due to disc herniation	48	45	Yoga and patient education program (Y+Edu; *n* = 24	Control group (CG; *n* = 24): Education programme	Y+Edu: 37.8 (6.0) CG: 38.2 (7.9)	100	12 weeks, 1 h, twice per week, 24 h of yoga in total	NR	Pain intensity (VAS 0–10 MPQ total), Functioning (ODI, Schober, PKE), Mood (MPQ Affective)
Zhu, 2019	Randomized controlled trial (2 arms)	China	Postherpetic neuralgia	50	50	Mindfulness-based stress reduction (MBSR; *n* = 25)	Control group (CG; *n* = 25): Usual care	MBSR: 55.2 (5.1) CG: 54.9 (4.6)	46.0	8 weeks, 1 per week, 150 min	Yes, at least 6 per week, at least 45 to 60 min	Pain intensity (NRS 0–10) Depression (HAMD-24) Anxiety (HAMA-14 0–56)

CPG: Chronic Pain Grade questionnaire; FACIT-F (Utrecht Activities List), and life satisfaction (Life Satisfaction Questionnaire).

The included studies were published between 2002 and 2022, and had an average dropout rate of 12.3%, which corresponds to 94 patients. In total, the combined study population included 764 patients with chronic neuropathic pain. The median age of patients in the intervention groups was 55.4 years, with an interquartile range (IQR) spanning from 54.3 to 61.0 years. In the control groups, the median age was 57.2 years, with an IQR of 52.5 to 60.9 years. The analysed sample sizes across these studies varied, ranging from 27 to 69 patients, and the percentage of female patients ranged from 6.4% to 100%.

The detailed rehabilitation interventions and control protocols as well as the examined outcomes are also summarized in Table I. Most common interventions were cognitive behavioural programmes including acceptance and commitment therapy (*n* = 4), mindfulness-based interventions (*n* = 5), and yoga (*n* = 2). The majority of control groups (*n* = 9) were inactive, including treatment as usual and waiting list. The mean total treatment duration of the 15 trials was 9.2 ± 4.9 weeks (median: 8 weeks; range: 4 to 24 weeks).

### Risk of bias assessment

**Fig. 2:**
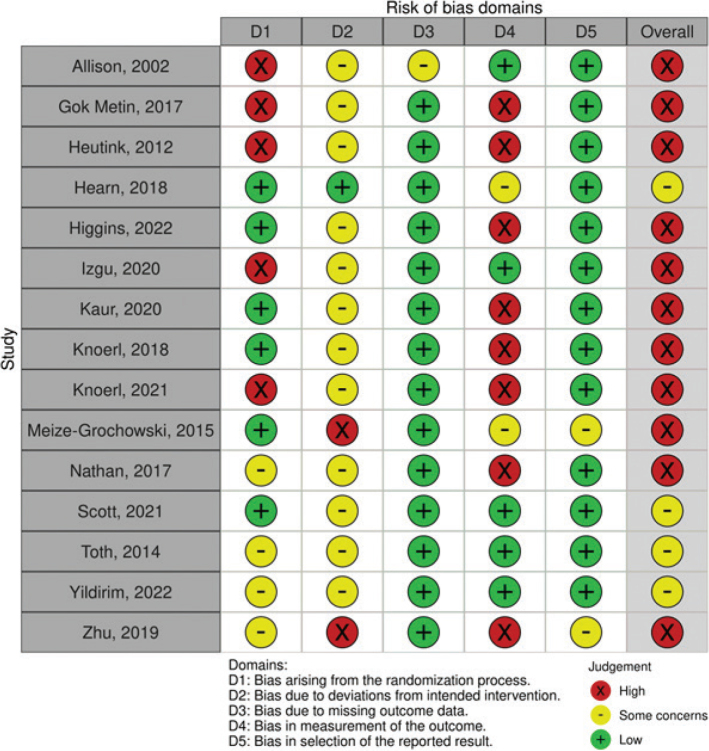
Risk of bias (RoB) plot.

Fig. 2 summarizes the risk of bias (RoB.2) assessments for individual domains and provides an overall assessment. Of the 14 studies, 4 (26.7%) were found to have some concerns, while 11 (73.3%) were rated as having a high risk of bias. It is important to note that, due to the nature of the treatment and control procedures, it was not possible for the initial authors to implement blinding for both therapists and patients in any of the included studies. Four studies were single-blinded (30, 38, 41, 42) while only in 3 studies (34, 40, 41) were outcome assessments carried out with blinding to the allocation (Fig. S1).

## OVERALL EFFECTIVENESS OF REHABILITATION INTERVENTIONS VERSUS CONTROLS

### Primary outcomes

**Fig. 3:**
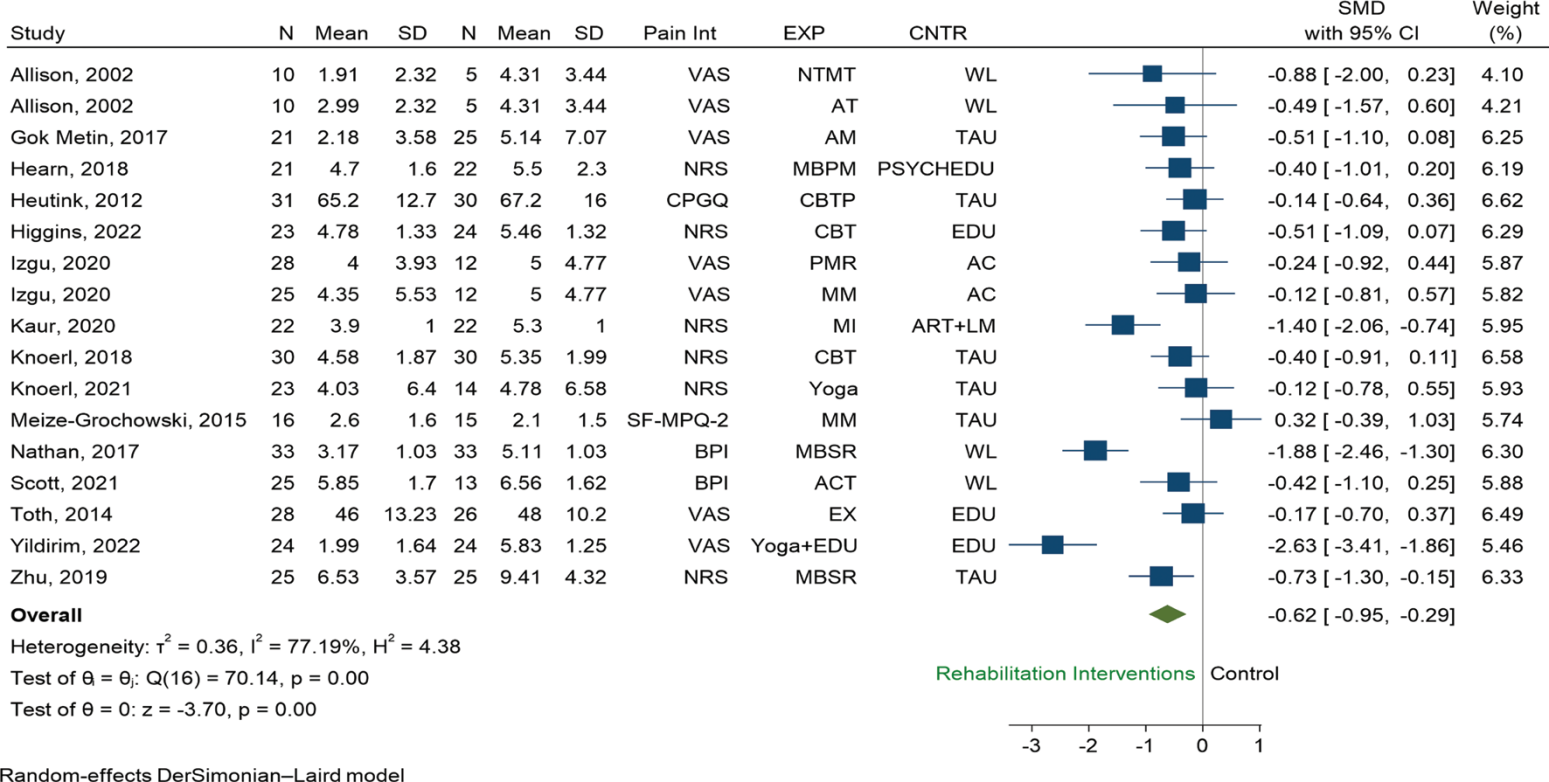
Random effect pair-wise meta-analysis of pain intensity effect sizes measured at short term: all rehabilitation interventions versus control. Abbreviations for the columns Pain Int (pain intensity), EXP (experimental group, i.e., type of rehabilitation intervention), and CNTR (control group), are as follows. Pain Int: BPI: Brief Pain Inventory, CPGQ: Chronic Pain Grade Questionnaire, NRS: numeric rating scale, SF-MPQ-2: Short-Form McGill Pain Questionnaire, VAS: visual analogue scale. EXP: ACT: acceptance and commitment therapy, AM: aromatherapy massage, AT: articular treatment, CBT: cognitive behavioural therapy, CBTP: cognitive behavioural treatment programme, EDU: patient education programme, EX: Exercise, MBPM: mindfulness based pain management, MBSR: mindfulness based stress reduction, MI: mental imagery, MM: mindfulness meditation, NTMT: neural tissue manual therapy, PMR: progressive muscle relaxation. CNTR: AC: attention control education, ART+LM: arithmetic tasks+listening to music, EDU: education, PSYCHEDU: psychoeducation online, TAU: treatment as usual, WL: waiting list.

*Pain intensity.* A total of 15 RCTs (*n* = 732) were eligible for pooling using random effects models, and results favoured the use of rehabilitation interventions over any control to reduce pain intensity (SMD –0.48, 95% CI –0.88 to –0.08, *p* = 0.02, I^2^ = 81.1%, Fig. 3). Restricted to studies using only psychological interventions, results favoured the use of psychological based interventions over any control to reduce pain intensity (SMD –0.49, 95% CI –0.88 to –0.10, I^2^ = 74.3%, 8 RCTs, *n* = 433, Fig. S2). Restricted to studies using other rehabilitation interventions than psychological, results also favoured the use of other rehabilitation interventions over any control to reduce pain intensity (SMD –0.79, 95% CI –1.38 to –0.20, I^2^ = 81.4%, 7 RCTs, *n* = 299, Fig. S2), but with greater reduction than psychological interventions.

**Fig. 4:**
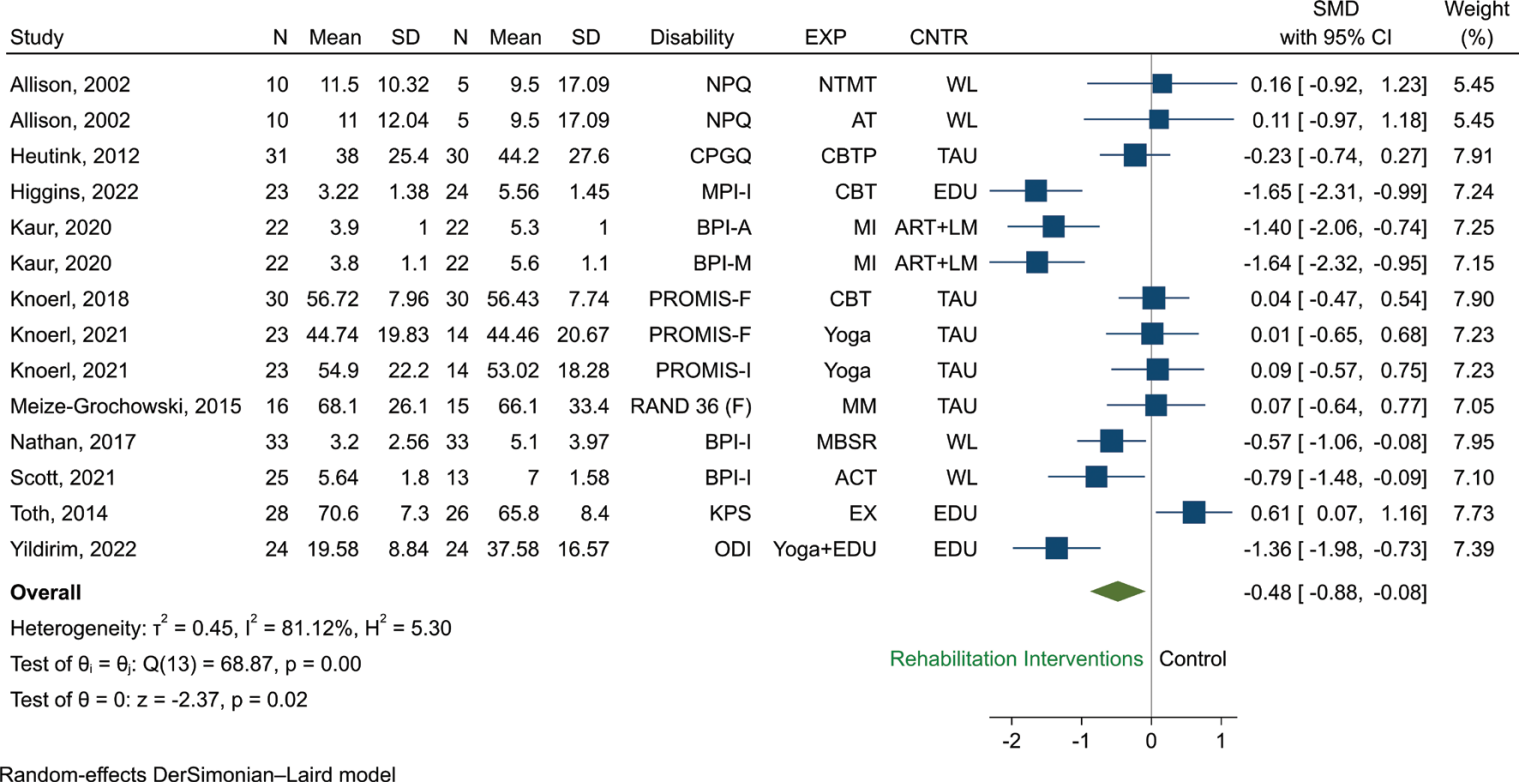
Random effect pair-wise meta-analysis of disability effect sizes measured at short term: All rehabilitation interventions versus control. Abbreviations for the columns Disability, EXP (experimental group, i.e., type of rehabilitation intervention), and CNTR (control group), are as follows. Disability: BPI-A: Brief Pain Inventory-Activity, BPI-I: , BPI-M: Brief Pain Inventory-Mobility, CPGQ: Chronic Pain Grade Questionnaire, KPS: Karnofsky Performance Scale, MPI-I: Multidimensional Pain Inventory-Interference, NPQ: Northwick Park Questionnaire, ODI: Oswestry Disability Index, PROMIS-F: Patient Reported Outcomes Measurement Information System-Functioning, PROMIS-I: Patient Reported Outcomes Measurement Information System-Interference, RAND 36(F): RAND 36(Functioning). EXP: ACT: acceptance and commitment therapy, AT: articular treatment, CBT: cognitive behavioural therapy, CBTP: cognitive behavioural treatment program, EDU: patient education programme, EX: exercise, MBSR: mindfulness based stress reduction, MI: mental imagery, MM: mindfulness meditation, NTMT: neural tissue manual therapy. CNTR: ART+LM: arithmetic tasks+listening to music, EDU: education, TAU = treatment as usual, WL = waiting list.

*Function or pain-related disability.* A total of 11 RCTs (*n* = 693) were eligible for pooling using random effects models, and results favoured the use of rehabilitation interventions over any control to reduce disability (SMD –0.45, 95% CI –0.85 to –0.05, *p* = 0.00, I^2^ = 83.9%, Fig. 4). Restricted to studies using only psychological interventions, results favoured the use of psychological based interventions over any control to reduce pain-related disability and improve function (SMD –0.51, 95% CI –0.98 to –0.03, I^2^ = 75.1%, 6 RCTs, *n* = 303, Fig. S3). Restricted to studies using rehabilitation interventions other than psychological, we found no statistically significant difference between other rehabilitation interventions vs any control (SMD –0.45, 95% CI –1.11 to 0.22, I^2^ = 85.6%, 5 RCTs, *n* = 294, Fig. S3).

*Work productivity.* A total of 2 RCTs (*n* = 82) were eligible for pooling using random effects models, and results favoured the use of rehabilitation interventions over any control to reduce work impairment (SMD –0.99, 95% CI –1.62 to –0.36, *p* = 0.00, I^2^ = 43.3%, Fig. S4).

*Publication bias for primary outcomes.* The assessment for publication bias was conducted specifically for pain intensity and function or pain-related disability outcomes due to the availability of more than 10 studies exclusively for these measures. As illustrated in Figs S5 and S6, the funnel plots displayed asymmetry solely for pain intensity and not for function or pain-related disability. Nevertheless, the Egger’s regression intercept analysis indicated the absence of publication bias for both primary outcomes (*p* = 0.52 and *p* = 0.76).

*Meta regression analysis for primary outcomes.* A comprehensive meta-regression analysis was undertaken to investigate potential modifiers’ effects on pain intensity, including variables such as the type of instrument used, the type of experimental interventions, control types, the RoB assessment, sample sizes, the specific type of neuropathic pain studied, and patients’ age. However, the findings revealed that none of these factors exhibited a discernible influence on the observed results (Fig. S7). This was also the case for function or pain-related disability (Fig. S8). These suggest that within the scope of this analysis, these factors did not contribute significantly to variations in the observed pain intensity results across the studies included.

### Secondary outcomes

*Psychological outcomes:* A total of 12 RCTs (*n* = 563) were eligible for pooling using random effects models for psychological outcomes including anxiety, depression, fatigue, and overall mood. The results favoured the use of rehabilitation interventions over any control to reduce mood dysfunction (SMD –1.57, 95% CI –2.31 to –0.82, *p* = 0.00, I^2^ = 76.4%, 3 RCTs, *n* = 158, Fig. S9). A total of 2 RCTs (*n* = 118) were also eligible for pooling using random effects models, and results favoured the use of mindfulness-based interventions over any control to reduce catastrophizing (MD –11.66, 95% CI –13.58 to –9.74, p = 0.00, I^2^ = 26.8%, Fig. S10). No other significant results were found.

*Quality of life.* A total of 8 RCTs (*n* = 391) were eligible for pooling using random effects models for quality-of-life (QOL) outcomes including physical, mental, environmental, and social components as well as overall QOL. The results favoured the use of rehabilitation interventions over any control to improve overall QOL (SMD 0.34, 95% CI 0.06 to 0.63, *p* = 0.02, I^2^ = 28.8%, 4 RCTs, *n* = 229, Fig. S11). No other significant results were found.

*Other outcomes.* A total of 7 RCTs (*n* = 341) were eligible for pooling using random effects models for other outcomes including pain acceptance, activities participation, satisfaction, and sleep problems. The results favoured the use of rehabilitation interventions over any control to improve sleep impairment (SMD –0.86, 95% CI –1.42 to –0.31, *p* = 0.04, I^2^ = 68.5%, 4 RCTs, *n* = 182, Fig. S12). No other significant results were found. One study also reported a non-significant effect on impression of change (OR 2.35, 95% CI 0.55 to 10.84, *p* = 0.21) (36).

### Summary of evidence

Table II shows the quality of evidence for each primary outcome (22). The evidence suggests a potential benefit of the rehabilitation interventions on pain intensity, function, and work productivity. However, the overall quality of the evidence was low to very low due to concerns about risk of bias, inconsistency, indirectness, and imprecision. Further research is likely to have an important impact on our confidence in these estimates.

**Table II:** Summary of evidence for the primary outcomes

Outcome	k	*n*	Summary ES (95%CI)	Risk of bias	Inconsistency	Indirectness	Imprecision	Publication bias	Quality level
Pain intensity	15	732	SMD, –0.48 (–0.88 to –0.08)	↓	↓	V	↓	V	Low
Function or pain-related disability	11	693	SMD, –0.45 (–0.85 to –0.05)	↓↓	↓	V	↓	V	Very low
Work productivity	2	82	SMD –0.99 (–1.62 to –0.36)	↓	↓	V	↓↓	NA	Very low

K: Number of RCTs; *n*: sample size; ES: effect size; SMD: standardized mean difference; NA: not applicable.

↓ = Downgrade by one level.

↓↓ = Downgrade by two levels. V = No downgrade.

## DISCUSSION

### Summary of main results

The main findings of this systematic review with meta-analysis were that rehabilitation interventions reduced short-term pain intensity (15 studies) and pain-related disability (11 studies) for patients with chronic neuropathic pain. Psychological interventions had effects on both pain intensity and pain-related disability, whereas other interventions had an effect on pain intensity but not on pain-related disability. Rehabilitation interventions also reduced work impairment (2 studies). However, at this stage, one should be cautious not to overemphasize the lack of evidence for other interventions on pain-related disability (see below in the subsection on quality of the evidence and implications for practice). All in all, rehabilitation interventions, particularly psychological methods, show promise in reducing pain intensity and disability in patients with chronic neuropathic pain. However, given the current limitations in the evidence, it is essential to conduct further research to establish the long-term benefits and comparative effectiveness of various rehabilitation approaches. Clinicians should consider a holistic and individualized approach to pain management, integrating multiple types of interventions to address the complex nature of neuropathic pain.

### Agreements/disagreements with earlier literature

The topic of rehabilitation interventions for chronic neuropathic pain has hitherto not been extensively covered in the literature. The importance of a rehabilitative stance, and the likelihood that such a stance will grow in importance, has been pointed out, as has the lack of evidence (15, 17). In 2015, Eccleston et al. published a systematic review on psychological therapies in neuropathic pain (14) and concluded that there is insufficient evidence regarding the efficacy and safety. One of the 2 papers included by Eccleston et al. is also part of our material (31). Of the 15 studies in the present study, only 3 have a publication date before 2015 (Table I), implying that the field has generated substantial material during the last decade. Hence, the present study has a high degree of novelty. Our findings are congruent with our recent registry-based real-life data study on 16,000 patients from the Swedish Quality Registry for Pain rehabilitation (SQRP), according to which neuropathic pain patients can benefit from IPRP (44) – although it has to be acknowledged that the validity of the neuropathic/non-neuropathic dichotomy is arguably the major limitation of that study. Grading the probability of neuropathic pain is a complicated matter (1), and achieving a high degree of diagnostic validity and reliability in a registry such as SQRP is very difficult. In this regard, we look forward to the implementation of ICD-11 (45). These limitations notwithstanding, in our opinion, it is important to relate findings from systematic reviews and meta-analyses to real-life evidence from registries such as SQRP, which is a nationwide, well-established registry for pain rehabilitation in Sweden.

### Completeness and applicability of evidence

The median age of patients in the intervention groups was 55.4 years vs 57.2 years in the control groups. Our study population seems to be older than the population of chronic pain patients included in SQRP, where the median age of almost 34,000 patients with chronic pain was 44 years (46). Concerning neuropathic pain patients, the mean age in a cohort from the same registry was 45 years (44). Hence, one could question the degree to which the material of the present study is representative of clinical practice when it comes to age, the patients being older than in SQRP data.

Concerning the distribution of sex, the studies we included in our material ranged from 6.4% to 100% women. Hence, there was great heterogeneity in this respect, making it difficult to generalize this aspect to clinical practice. In SQRP as a whole, 72% of patients are female (46), whereas the corresponding figure for neuropathic pain is 59% (44).

Moreover, in the present material, 7 out of 15 studies concerned some form of painful polyneuropathy. In an SQRP study, however, only less than 1% of diagnoses deemed compatible with possible neuropathic pain had an ICD-10 diagnosis of polyneuropathy (G62.9). In this case, given how common diabetic polyneuropathy is, we think that this is indicative of a bias in referral of patients with polyneuropathy to SQRP, i.e., that the population of the present material is closer to chronic neuropathic pain diagnoses “reality” than SQRP in this respect. In other words, it seems patients with painful polyneuropathy are very rarely referred to a clinical department delivering IPRP in Sweden.

Concerning the interventions, most of them were related to cognitive behavioural therapy, and we therefore think our results are the most applicable when it comes to psychological interventions. Importantly, however, in contrast to our plan and because of lack of data, we did not analyse long-term effects but instead had to focus on short-term outcomes, i.e., on the assessment point immediately following the intervention or at the first-time measurement conducted after the intervention period. Hence, our findings are not applicable to the question of long-term effects. Also, it is worth pointing out that we did not define electrostimulation or magnetic stimulation techniques as rehabilitation interventions.

### Quality of the evidence and implications for practice

This systematic review and metanalysis shows that there is evidence for short-term effects of rehabilitation interventions in patients with chronic neuropathic pain. While psychological interventions had effects on pain intensity and function, other interventions including physical activity did not have a significant effect on function. However, the overall quality of evidence is low and there is a lack of sufficient studies assessing the long-term effects of different rehabilitation interventions. The majority of included studies had a high risk of bias. Concerning publication bias, despite asymmetry in the funnel plot for pain intensity, we did not find any indication of publication bias for primary outcomes.

When it comes to psychological outcomes including anxiety, depression, fatigue, and overall mood, the results favoured the use of rehabilitation interventions to reduce mood dysfunction. Pain catastrophizing is considered to be one of the most robust predictors of adverse pain outcomes and its association with pain severity has been reported in experimental and clinical studies, in response to a wide range of medical procedures and in every pain population in which it has been studied, cross-culturally (47). Yet, we found only 2 eligible RCTs (*n* = 118) reporting pain catastrophizing as an outcome of rehabilitation interventions in neuropathic pain.

Pain acceptance, activities participation, satisfaction, and sleep problems were outcomes in a total of seven eligible RCTs (*n* = 341). The results favoured the use of rehabilitation interventions over any control to improve sleep impairment only and no other significant results were found. One study also reported a non-significant effect on impression of change.

Although several of the 15 included papers studied interventions that are part of IPRP (i.e., components of IPRP), none of the papers had a complete IPRP as an intervention. Based on the biopsychosocial model of pain, IPRP is recommended to patients with treatment resistant chronic pain. These programmes are mainly based on education, exercise therapy, work interventions and cognitive behavioural therapy (CBT), including acceptance and commitment therapy (ACT), addressing factors such as catastrophizing, pain acceptance, and psychological flexibility (48). This treatment has traditionally been more available for patients with non-neuropathic pain than those with neuropathic pain. In a recent study we looked at the real-world effects of IPRP on patients with chronic neuropathic pain compared with non-neuropathic patients using patient-reported outcome measures (PROMs) available in SQRP (44), and we showed that IPRP yielded equal or in some cases slightly superior outcomes for neuropathic pain, with no differences between the groups before the intervention. Cognitive-emotional factors are arguably of great importance in any type of pain, and we suggest that available evidence is in line with the clinical experience that different subgroups of chronic pain patients might have the same extent of burden and suffering regardless of pain mechanism (nociceptive, neuropathic, nociplastic) (49). However, in this metanalysis, acceptance, activity participation, and impression of change, which in clinical practice are believed to be important factors for the outcome of IPRPs, were not improved.

Alongside postulated central changes, aberrant primary afferent activity and chronic, low-grade, systemic inflammation might remain as an important nociceptive drive in some chronic pain states (50). Further elucidation of the differential impact of central and peripheral changes in neuropathic pain conditions is needed. This would improve clinicians’ decision-making when choosing the type and the timing of different therapeutic interventions (i.e., pharmacological, invasive, and rehabilitation interventions).

The term “rehabilitation potential” refers to assessment of the probability that a person who receives rehabilitation will show improvement in outcomes. There is, however, a lack of definition and clinicians’ assessment of patients’ “rehabilitation potential” can be influenced by geography, local service models, and patient-related social and personal factors. Furthermore, rehabilitation is not a single intervention. It is a systematic complex step-by-step process/intervention, taking the approach of trial and error, with the aim of improving the different problems a person may have. Integrating evidence gained from RCTs, systematic reviews, and metanalysis with real-world data as well as qualitative approaches will improve our knowledge of what benefits each individual patient and will in the long run help us develop better rehabilitation interventions for patients with neuropathic pain.

### Strengths and limitations

First, our study has comprehensive coverage. The inclusion of various intervention types enhances the breadth and depth of our findings, providing valuable insights into the multifaceted nature of neuropathic pain treatment. Second, the study specifically includes patient groups that are frequently encountered in clinical settings, like those suffering from painful polyneuropathy, i.e., there is a clear link to clinical practice. By focusing on this patient group, our study directly addresses a gap in current research, offering practical implications for the treatment of a condition that is common yet often challenging to manage effectively.

The study has several limitations. One is the age disparity of patients, with median ages in the intervention and control groups being higher (55.4 and 57.2 years respectively) compared with the 44 years median age of chronic pain patients in SQRP. There was also considerable heterogeneity in gender distribution across the studies, with the percentage of female patients ranging from 6.4% to 100%. This raises concerns regarding the representativeness of the included RCTs population. A further limitation is related to the possibility of a lack of diagnostic specificity concerning chronic neuropathic pain. Hence, it is conceivable that a proportion of the study population had non-neuropathic pain. Future studies should consistently use the grading system proposed by Finnerup et al (1). Another significant limitation is the focus of the included studies, particularly the emphasis on painful polyneuropathy, which is less common in SQRP. This indicates a potential referral bias in Swedish clinical settings. Additionally, the study’s main focus on cognitive behavioural therapy interventions implies that results are more applicable to psychological treatments. A critical limitation is the study’s exclusive focus on short-term outcomes. Consequently, the long-term effects of the interventions remain unaddressed, highlighting a significant gap in the study’s conclusions. The overall evidence quality is low, primarily due to the lack of long-term data and high risk of bias in most included studies. Although rehabilitation interventions showed benefits in improving certain psychological outcomes and sleep impairment, no other significant results were found, underscoring the need for more comprehensive research in this area.

### Implications for research

We had to exclude many papers that had “neuropathy” as an inclusion criterion and not “neuropathic *pain”*. Hence, it is of paramount importance that researchers conducting RCTs on rehabilitation interventions in these conditions be more precise when stating the inclusion criteria. All in all, although much has happened since the paper of Eccleston and co-workers in 2015 (14), more research is needed concerning rehabilitation interventions and their effect on chronic neuropathic pain.
